# Vaccine Delivery Methods into the Future

**DOI:** 10.3390/vaccines4020009

**Published:** 2016-03-28

**Authors:** Vasso Apostolopoulos

**Affiliations:** Centre for Chronic Disease, College of Health and Biomedicine, Victoria University, Melbourne, VIC 3021, Australia; vasso.apostolopoulos@vu.edu.au; Tel.: +61-3-9919-2025; Fax: +61-3-9919-2465

**Keywords:** vaccine, vaccine delivery, antigen delivery, immunotherapy

## Abstract

Several modes of vaccine delivery have been developed in the last 25 years, which induce strong immune responses in pre-clinical models and in human clinical trials. Some modes of delivery include, adjuvants (aluminum hydroxide, Ribi formulation, QS21), liposomes, nanoparticles, virus like particles, immunostimulatory complexes (ISCOMs), dendrimers, viral vectors, DNA delivery via gene gun, electroporation or Biojector 2000, cell penetrating peptides, dendritic cell receptor targeting, toll-like receptors, chemokine receptors and bacterial toxins. There is an enormous amount of information and vaccine delivery methods available for guiding vaccine and immunotherapeutics development against diseases.

## 1. Introduction

The first attempts to prevent infectious diseases was over 1000 years ago in China, whereby contents of smallpox vesicles were injected into people who had not previously experienced smallpox. Fatalities were uncommon in the individuals inoculated with the smallpox vesicles, compared with victims of natural smallpox infection. More than 700 years later, Edward Jenner injected an 8 year old boy with cowpox and challenged him with smallpox, the boy was subsequently protected against smallpox. Hence the term “cross reactivity” was coined. Two hundred years later, smallpox vaccination became increasingly popular in the decade 1967–1977 and complete world-wide eradication was accomplished. Remarkably, the Jennerian approach to immunoprophylaxis remains valid. It should be noted that Louis Pasteur, himself one of the great vaccinologists, coined the term “vaccine” in honor of Jenner, to refer to immunizing agents. 

Numerous methods of vaccination have been used, such as: attenuated bacteria, live viruses, dead organisms and despite their success, a number of disasters in humans have resulted. Disasters were primarily due to improper lab manufacturing and handling and consequently these incidences led to improved procedures and the safety of vaccines, and led to regulatory measures to assure proper laboratory conditions. With attempts to control more complex diseases and the need to improve vaccine safety, stability, efficacy and cost, there is pressure for precisely defined vaccines. 

Public awareness of health and safety issues vaccines must now meet higher standards of safety and biochemical characterization than they did in the past. Some of the vaccines developed in the past would not even meet the minimum standards required today. Hence, new improved precisely defined highly purified vaccines need to be developed. Advances in fields such as peptide synthesis, molecular biology, protein production, crystallography, cell biology, immunology and animal models are required for the development of new and improved vaccines, in an attempt to move from traditional live virus vaccines to the theoretical safer but “less immunogenic” vaccines. In an attempt to improve the immunogenicity of the highly purified vaccines, a number of technologies and delivery methods have been developed over the years, in order to deliver antigens to induce appropriate responses. 

## 2. Vaccination

**Figure 1:**
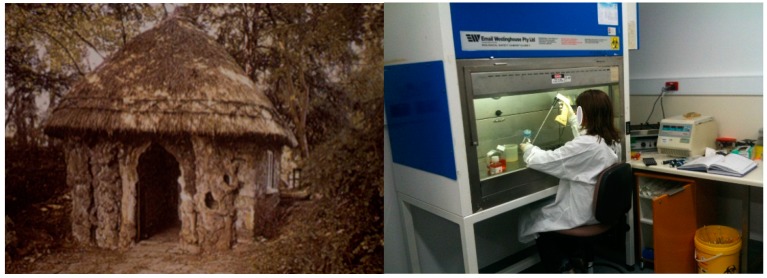
Working conditions of Edward Jenner, “Jenner’s Hut” (**left**) used to inject people against smallpox, compared to working conditions today for developing new vaccine delivery methods (**right**).

The biggest triumph in the history of vaccination, was the eradication of smallpox, and it was achieved without a specific delivery vehicle, and without detailed knowledge of immune cell activation. Interestingly, Edward Jenner injected people with cowpox vesicles at the back of a hut at his home, “Jenner’s hut”, where no, good laboratory practice (GLP) and good manufacturing practice (GMP) regulations were in place (Figure 1). Today, the immunizing antigen must be clearly defined, can’t be a crude extract, epitopes identified, a delivery method used, evidence that the vaccine induces an immune response in pre-clinical models. For a vaccine to be used and injected into an individual, endless amounts of paperwork are required and vaccines must meet GLP and/or GMP quality and clinical trials to be conducted under good clinical practice (GCP) guidelines. How many of the vaccines produced today, have eradicated a disease? Is it because safer and more precise vaccines are required? Or is it, because no worldwide mass vaccinations have occurred, since smallpox? Perhaps the public awareness and regulations put in place in most countries for compulsory injections of measles, mumps, rubella (MMR) vaccinations in infants and chickenpox vaccine in toddlers, may lead to their eradication within the next generation.

## 3. Technologies Used in the Generation of New Improved Vaccines

The application of genetic and recombinant DNA approaches to vaccination has led to new possibilities of safer and more efficient vaccines. Recombinant DNA technology can be applied to antigen identification and isolation, and by being able to clone and express all the antigens of an organism individually, overcomes two major hurdles associated with traditional vaccines. First, before the recombinant DNA era, it was difficult to obtain sufficient quantities of particular antigens in a pure enough form to allow the appropriate testing. Recombinant DNA technology overcame this problem, and second, recombinant DNA technology has made the study of pathogenic organisms safer because single genes and their translation products are examined rather than the whole organism [1,2,3]. 

Synthetic peptide chemistry has greatly contributed to vaccinology, where peptides (2–100 amino acids) could be synthesized and used as immunogens, in addition to synthesizing T and B cell epitopes and used as vaccines. Nuclear magnetic resonance (NMR), molecular modelling and X-ray crystallography approaches in understanding protein structures has contributed enormously into the generation of improved vaccines. Numerous peptide based vaccines have been shown effective in pre-clinical and in human clinical trials [4,5,6,7]. The advent of these technologies stimulated the production of new vaccines and the identification of precise epitopes on antigens has made synthetic peptide vaccines a real possibility. Such vaccines are designed to be safer and more efficient. Unfortunately, there are still many obstacles for their clinical use; the limited immunogenicity of many of these candidates has hindered their development as potential vaccines. Strategies to enhance the immunogenicity of candidate vaccines are therefore required.

## 4. Methods of Vaccine Delivery: Approaches to Enhancing Immunogenicity

Adjuvants have the ability to amplify either or both humoral and cellular immune responses to an antigen. The most commonly used adjuvant in experimental animals is complete and incomplete Freund’s adjuvant, and, although very effective in evoking effective and long lasting immune response, it is not suitable for human use. There is only one registered human adjuvant (aluminum hydroxide or aluminum phosphate) which is used in diphtheria, tetanus and hapatitis B vaccines. Aluminum salt adjuvants are limited in their use, in that they preclude lyophilisation or freezing, they are not effective with all antigens and they do not stimulate cell-mediated immunity. Candidates for alternative adjuvants for vaccine development, include, the Syntex formulation, SAF-1 (containing squalene oil, an amino acid derivative of muramyl dipeptide (threonyl-MDP) and nonionic block polymers), Ribi formulation (containing mycobacterial cell walls and monophosphoryl lipid A) and saponin derivative, QS21 [8]. 

Liposomes (phospholipid-based vesicles) were extensively used in the 1970s to deliver or target drugs to specific sites in the body. An additional promising aspect of the system is its immunoadjuvant action, shown to induce humoral and/or cell-mediated immune responses for liposome-entrapped antigens with or without cytokines or other immunological active agents [9]. Another approach involves incorporation of antigens into solid particles called ISCOMs (immunostimulatory complexes). ISCOMs are composed of the adjuvant Quil A and peptides. ISCOM particles contain low concentrations of adjuvant and can therefore significantly enhance immunogenicity of an antigen [10,11]. 

The multiple antigen peptide approach (MAP), also known as dendrimers, is aimed at replacing a protein carrier in a peptide based vaccine, with a small structural unit that can amplify peptide antigens without the disadvantages associated with protein carriers. The framework of the MAP is a core matrix consisting of branching trifunctional amino acids (such as, lysine) with the following properties: (i) non-immunogenic; (ii) ability to amplify the peptide antigens into a macromolecule; (iii) flexibility to incorporate multiple epitopes and (iv) accessible for chemical synthesis. MAP (dendrimer) technology has been used to successfully induce immune responses to antigens [12,13].

The ideal vaccine for many diseases is a live attenuated derivative of the pathogen which can induce protective immunity to antigens on the pathogen without causing any side effects. Barriers to the development of such vaccines, however, give rise to difficulties in growing the pathogen in the laboratory and difficulties in attenuating the pathogen. One strategy of overcoming these barriers is to insert the pathogen’s genes into a nonpathogenic organism. The non-virulent organism (recombinant virus) serves as a vector for the expression of the genes coding for the antigens. This has the advantage that the recombinant virus simultaneously synthesizes the foreign antigen and delivers it to the host’s immune system. Several vectors have been tested in the development as vaccines, such as, vaccinia virus, vaccinia virus ankara, avipoxvirus, adenovirus, alphavirus and enterovirus vectors [14,15].

Immunization with non-replicating plasmid DNA (“naked DNA”) encoding viral proteins may be advantages to use as vaccines, because no infectious agent is involved, there is no requirement for assembly of virus particles and determinant selection is permitted. In animal models strong immune responses were generated; however, these did not translate into human clinical trials. As a result delivery methods for DNA were required, and as such, *in vitro* and *in vivo* electroporation, gene-gun and Biojector 2000 were developed. Other modes of delivery include, non-viral vectors and receptor mediated uptake of DNA into cells [16,17].

Cell penetrating peptides (CPP) or membrane translocating peptides (MTP) are a group of cationic peptides that have the ability to enter into the cytoplasm of cells. CPP are able to deliver a number of antigens, including RNA, DNA, peptides, proteins, drugs and virus particles into cells. In fact, CPP (i) TAT from the human immunodeficiency virus transactivator of transcription protein and (ii) penetratin from the *Drosophila Antennapedia* domain have been used to deliver antigens with strong cellular and antibody response induction[18].

In addition, an array of other methods of vaccine delivery have shown great promise in animal models and in human clinical trials. Such methods include, use of nanoparticles, virus-like particles, use of *ex vivo* grown monocyte derived dendritic cells and targeting antigens to dendritic cell receptors such as, c-type lectin receptors, mannose receptor, DC-SIGN, DEC205, L-SIGN, DC-SIGNR, CIRE, FIRE, Langerin, MGL, dectin-1, dectin-2, DNGR-1, Clec12A, Clec12B, Clec2, LOX1, DCIR, scavenger receptor, F4/80 receptor, DC-STAMP and Fc receptor [19,20]. More recently, there has been an upsurge of information regarding toll-like receptor (TLR) targeting and stimulation of DCs via TLR. In mice, TLR ligands activate DCs and elicit immune responses [21,22,23], however, no substantial TLR-targeting vaccine trials have been completed in humans and it is unknown whether TLR targeted approach will result in significant benefits in humans as those seen in mice. Moreover, targeting antigens to chemokine receptors on DCs (CCR1, CCR2, CCR5, CCR6, CXCR1, CXCR4) have shown to generate strong immune responses *in vitro* and *in vivo.* Furthermore, bacterial toxins and DC binding peptides target antigens to DCs without the requirement of DC receptor targeting [20]. In the special issue of “Vaccine delivery”, the latest methods of, live-attenuated bacterial vectors [24], nanoparticle based vaccines [25,26], synthetic carriers [27], cholera toxin subunit B as an adjuvant [28] and Gavi vaccination programs [29], are extensively reviewed.

## 5. Conclusions

Over the last 25 years, vaccine delivery methods have been developed to induce immune responses to the highly purified and ‘safe’ vaccines. Numerous methods induce strong cellular, humoral and/or protection in animal models, and are rapidly moving into human clinical trials. We await the outcomes that will show which vaccine delivery method will ultimately prove useful in eradicating disease.
